# Accelerating clinical development of HIV vaccine strategies: methodological challenges and considerations in constructing an optimised multi-arm phase I/II trial design

**DOI:** 10.1186/1745-6215-15-68

**Published:** 2014-02-26

**Authors:** Laura Richert, Adélaïde Doussau, Jean-Daniel Lelièvre, Vincent Arnold, Véronique Rieux, Amel Bouakane, Yves Lévy, Geneviève Chêne, Rodolphe Thiébaut

**Affiliations:** 1 Université Bordeaux, ISPED, Centre INSERM U897-Epidemiologie-Biostatistique, Bordeaux, F-33000, France; 2 INSERM, ISPED, Centre INSERM U897-Epidemiologie-Biostatistique, Bordeaux, F-33000, France; 3 Vaccine Research Institute (VRI), Créteil, F-94010, France; 4 CHU de Bordeaux, Pole de sante publique, Bordeaux, F-33000, France; 5 INRIA SISTM, Talence, F-33405, France; 6 INSERM U955, Créteil, F-94010, France; 7 Université Paris Est Créteil, Faculté de Médecine, Créteil, F-94010, France; 8 Groupe Henri-Mondor Albert-Chenevier, Immunologie clinique, Créteil, F-94010, France; 9 French National Agency for Research on AIDS and Viral Hepatitis (Inserm-ANRS), Paris, F-95013, France; 10 Centre INSERM U897, Université Bordeaux, 146, rue Léo Saignat – case 11, Bordeaux cedex, F-33076, France

**Keywords:** Clinical trial, Design, Phase I, Phase II, Randomisation, Frequentist, Bayes, Stopping rule, Safety, HIV vaccine

## Abstract

**Background:**

Many candidate vaccine strategies against human immunodeficiency virus (HIV) infection are under study, but their clinical development is lengthy and iterative. To accelerate HIV vaccine development optimised trial designs are needed. We propose a randomised multi-arm phase I/II design for early stage development of several vaccine strategies, aiming at rapidly discarding those that are unsafe or non-immunogenic.

**Methods:**

We explored early stage designs to evaluate both the safety and the immunogenicity of four heterologous prime-boost HIV vaccine strategies in parallel. One of the vaccines used as a prime and boost in the different strategies (vaccine 1) has yet to be tested in humans, thus requiring a phase I safety evaluation. However, its toxicity risk is considered minimal based on data from similar vaccines. We newly adapted a randomised phase II trial by integrating an early safety decision rule, emulating that of a phase I study. We evaluated the operating characteristics of the proposed design in simulation studies with either a fixed-sample frequentist or a continuous Bayesian safety decision rule and projected timelines for the trial.

**Results:**

We propose a randomised four-arm phase I/II design with two independent binary endpoints for safety and immunogenicity. Immunogenicity evaluation at trial end is based on a single-stage Fleming design per arm, comparing the observed proportion of responders in an immunogenicity screening assay to an unacceptably low proportion, without direct comparisons between arms. Randomisation limits heterogeneity in volunteer characteristics between arms. To avoid exposure of additional participants to an unsafe vaccine during the vaccine boost phase, an early safety decision rule is imposed on the arm starting with vaccine 1 injections. In simulations of the design with either decision rule, the risks of erroneous conclusions were controlled <15%. Flexibility in trial conduct is greater with the continuous Bayesian rule. A 12-month gain in timelines is expected by this optimised design. Other existing designs such as bivariate or seamless phase I/II designs did not offer a clear-cut alternative.

**Conclusions:**

By combining phase I and phase II evaluations in a multi-arm trial, the proposed optimised design allows for accelerating early stage clinical development of HIV vaccine strategies.

## Background

Although several promising approaches to prevent human immunodeficiency virus (HIV) infection have been put forth in the recent years [1-4], the development of a prophylactic vaccine strategy remains a key goal in the effort to end the HIV epidemic.

The main prophylactic HIV vaccine candidates currently under study include subunit or epitope-based vaccines with or without adjuvants, recombinant virus-vector vaccines and deoxyribonucleic acid (DNA) vaccines. The combination of different vaccines into heterologous prime-boost strategies is today considered a promising approach. Indeed, results of a phase IIB trial in Thailand (RV144 trial), combining a virus-vector vaccine prime with a subunit boost, showed a modest protective effect of the tested HIV vaccine strategy [5]. HIV vaccine development remains ongoing and too lengthy. For instance, clinical development of the components of the HIV vaccine strategy evaluated in the RV144 trial started in the mid-1990s in Thailand, however the results of the aforementioned phase IIB trial only became available in 2009. According to development timelines, a confirmatory phase III trial with an improved vaccine strategy is not projected to start before 2019 [6,7].

The fact that immunological correlates of protection are not well understood has hampered HIV vaccine development to date [8]. A protective vaccine effect can currently not be predicted on the basis of vaccine-induced immunogenicity markers. Since transition from phase I/II to phase IIB/III trials can thus not rely on a validated surrogate immunogenicity endpoint, and large sample sizes and resources are required for later-stage trials with HIV-acquisition endpoints (phase IIB/III), the clinical development of HIV candidate vaccine strategies is laborious. Phase I and II HIV vaccine trials usually include extensive immunogenicity assessments, measuring both the humoral and cellular immune responses to vaccines with different markers and techniques. All of the above complicates the decision to set up a large-scale IIB/III trial, powered for an HIV-acquisition endpoint. These constraints have resulted in only six HIV vaccine phase IIB/III trials conducted so far, five of which yielded disappointing results without any evidence for protective vaccine effects [9-11].

Recently, calls for accelerated clinical development of HIV vaccine strategies have been put forward, advocating the implementation of adaptive trial designs for this purpose [12,13]. Indeed, numerous adaptive or multi-stage clinical trial designs for different phases of clinical development are available in the methodological literature. However, these designs were mainly devised for cancer treatment trials, which differ from HIV vaccine trials. In oncology, the patients who are enrolled in trials suffer severe illness requiring treatment. Inefficacious strategies must thus be identified as soon as possible to avoid harm. In contrast to oncology trials, including trials for therapeutic cancer vaccines [14], the development of prophylactic HIV vaccines targets a non-ill population and thus necessitates specific design considerations. Moreover, assessment of endpoints is clearer in oncology trials both because of a higher incidence and more well-defined surrogate endpoints, such as tumour response criteria [15]. The application of adaptive trial designs to HIV vaccine research is challenging given that numerous immunogenicity endpoints without a definite hierarchy are evaluated in phase II trials and that clinical development plans should remain flexible enough to accommodate new scientific knowledge. Thus, trial design optimisation has rarely been considered in HIV vaccine research, yet as the number of potential HIV vaccine strategies under development increases it has become of greater interest [16]. Gilbert et al. proposed an adaptive phase IIB/III design with an HIV-acquisition endpoint for mid- to late-stage clinical development (phase IIB/III), which has yet to be applied in practice [17]. For early-stage development, Moodie et al. recommended a phase IB selection design in 2006 to prioritise among different candidate vaccine strategies the one with the top-rank based on an immunogenicity endpoint. At that time, they considered that knowledge about HIV vaccine immunogenicity was too limited to select vaccine strategies using bounds based on a theoretical response rate [18]. To date, although understanding of vaccine-elicited immune responses still requires much more progress, this justification has evolved. We now consider it purposeful to define an insufficient response level in a widely used, validated immunogenicity screening assay [19].

Currently, more than 20 ‘generic’ HIV candidate vaccines are in early clinical development [16]. Given that generic candidates may be tested with different antigen inserts, that combinations of different candidate vaccines are evaluated and that strategies with the same combination may differ in terms of the timing of injections, the number of potential vaccine strategies is large. Efficient screening of potential strategies and decision making in early stage development is thus crucial. In the present article, we present an early-stage trial design, integrating phases I and II to evaluate both safety and immunogenicity endpoints of several HIV vaccine strategies in parallel and allowing for the rapid cessation of those with insufficient safety or immunogenicity levels.

## Methods

### Motivating example

Our search for an optimised phase I/II HIV vaccine trial design was motivated by the clinical development plan of a vaccine portfolio comprising three prophylactic HIV candidate vaccines, referred to herein as ‘vaccine 1’, ‘2’ and ‘3’.

Vaccine 1 is a viral vector (modified vaccinia Ankara (MVA) virus) expressing HIV antigens, which has not yet been evaluated in humans with the specific HIV inserts used. It therefore requires a phase I safety study before it can be administered to a larger number of volunteers. However, the MVA vector with different HIV and non-HIV inserts has already been studied in previous trials and has a good safety profile in humans [20-26]. Minimal toxicity is thus expected for vaccine 1. The tested target dose (1x10^8^ plaque-forming units) corresponds to the standard dose tested for MVA vaccinations against smallpox [22,27] and to the dose selected in previous dose-escalation studies of MVA vectors with HIV and non-HIV inserts [20,24,28,29]. No dose escalation is thus planned for this vaccine in our development plan.

Vaccine 2 is a lipopeptide vaccine, including an equal-weight mix of five synthetic HIV-1 peptides coupled to a palmytoil tail (HIV LIPO-5). Vaccine 3 is a deoxyribonucleic acid (DNA) vaccine, encoding for a multi-HIV antigen. Both vaccines 2 and 3 have been studied previously in phase I and II trials and have a good safety record [30-33].

**Figure 1:**
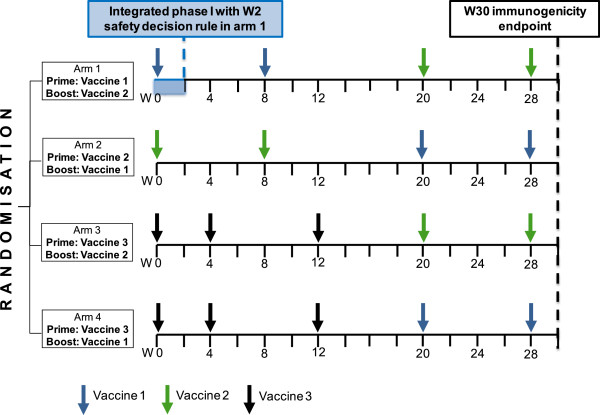
**Overview of the proposed multi-arm phase I/II design.** Legend: W = week.

In the clinical development plan of this vaccine portfolio, the three vaccines are combined into four heterologous (that is, using different vaccines in the prime and the boost phase) prime-boost strategies (Figure 1). Vaccine 3 is currently being considered only for use as a prime vaccine component, and not as a boost [34].

In our clinical development plan, the aim of the phase I evaluation of vaccine 1 at the target dose is to stop administrations of this vaccine as soon as possible in case of a safety problem. The early stage of the phase II evaluation aims at screening each of the four prime-boost combinations for a minimum immunogenicity level. Non-immunogenic or unsafe vaccine strategies should be discarded as soon as possible. A ‘classical’ clinical development scheme with several phase I and II trials to address these objectives would be a lengthy process. In order to accelerate the clinical development and given the high likelihood of a good safety profile of vaccine 1 similar to the one observed with different HIV inserts, we sought to set up an optimised trial design combining phases I and II for evaluation of the four prime-boost strategies in parallel.

For the purpose of the present work, we defined an ‘optimised design’ as a trial design allowing for a gain in timelines (and, if possible, in the number of trial participants) compared to separate phase I and II trials, while remaining feasible and respecting the requirements outlined below.

### Requirements for the optimised phase I/II vaccine trial design

In searching for an optimised design appropriate for our development plan, we imposed the following methodological requirements:

• First, a randomised multiple arm design is desirable to ensure comparability of populations and unbiased assessment of several vaccine strategies in parallel, even if no direct statistical comparisons are made between arms [35].

• Second, the design requires an evaluation of both immunogenicity and safety endpoints, assessed at different time points, allowing for an early safety decision rule to emulate the phase I evaluation of vaccine 1 during the vaccine prime phase. The dose used for vaccine 1 is considered well below the toxic dose.

• Third, endpoints for immunogenicity and safety are not necessarily correlated, since severe vaccine-related toxicity events and immunogenicity are likely to be unrelated [36].

• Fourth, an important constraint of the design is the fact that safety and immunogenicity outcomes are not observed simultaneously, since the immunogenicity measurements of all participants are planned in batch at a central laboratory at the end of the trial to allow for blinded assessments and to limit measurement error due to time and batch effects.

• Fifth, data integration of phase I into phase II evaluation should be possible to allow for an optimal use of the collected data.

• Sixth, the trial design must be compatible with high early accrual dynamics and with conduct as a multi-centre trial. Prophylactic HIV vaccine trials enrol healthy volunteers, a process that allows for better planning and management of recruitment dynamics than the enrolment of ill patients. To keep costs low, a single call for volunteers is planned for our trial. As previous vaccine trial recruitment studies have shown that a ‘waiting time’ between the first contact and enrolment may lead to a loss of interest in participation [37], the time between the call for volunteers and the start of the trial will be kept short. Altogether, this is expected to result in high early accrual dynamics.

• Lastly, a design without a control group was favoured since HIV-specific immune responses can be considered to be close to zero in healthy volunteers at low risk of HIV infection, as attested by immune response measurements in prophylactic HIV vaccine trials at baseline or in a placebo arm [20,21,38-40]. A vaccine strategy meeting the immunogenicity endpoint can therefore be deemed clearly distinct from a potential control group. Given that the volunteers in the planned trial will have a low-risk profile (restricted by the eligibility criteria) and the fact that all participants will receive an experimental vaccine strategy, we considered it unlikely that differential risk behaviour during the trial would have an impact on the immunogenicity endpoint.

A summary of these requirements is shown in the list below:

▪ randomized multi-arm trial

▪ evaluation of both efficacy and safety endpoints, with:

– early safety decision rule

– difference in timing of the evaluation between the two endpoints

– assumption that toxicity events and efficacy are likely unrelated

– efficacy outcome not observed in real-time (measured in batch in central lab at end of the trial)

– data integration of phase I into phase II efficacy evaluation

▪ compatibility with high early accrual dynamics and with conduct as a multi-centre trial

▪ no control arm

Moreover, although not a formal design requirement, an open-label design was preferred since the immunogenicity endpoint can be measured objectively and laboratory staff performing the measurements centrally will be blinded to trial arm and time point. Although a blinded control group would limit bias in the evaluation of safety, implementation of blinding is difficult in practice: indeed, blinding would require the introduction of dummy vaccine injections in each trial arm, since vaccination time points during the prime phase vary between vaccine strategies (Figure 1). Additional immunogenicity sampling time points after each dummy shot would then be necessary for maintaining blinding, requiring large amounts of blood. Blinding participants and site staff was thus not pursued for this trial design, but we acknowledge that a blinded placebo control arm could be helpful for safety assessments when feasible.

### Search for the optimised design

We first searched the literature for existing trial designs fulfilling the requirements outlined above in order to be appropriate for application to early stage clinical HIV vaccine development. We performed a literature search in Pubmed/Medline using the search terms (‘Clinical Trials, Phase II as Topic’ [MAJR]) AND (‘Therapeutics/adverse effects’ [MeSH] OR safety OR toxicity). In addition, the review and manual of phase II designs by Brown et al. were used to identify potential designs for our context [41]. Moreover, we reviewed the reference lists of the most relevant articles.

We then adapted a randomised phase II design to include an early safety decision rule during the prime phase of vaccine 1. We evaluated two types of statistical approaches for the safety decision rule: (1) a fixed-sample rule from a frequentist perspective; and (2) a continuous safety monitoring approach from a Bayesian perspective. Simulation studies were built to evaluate the statistical properties of the design with either decision rule. For each simulation scenario under different underlying safety and immunogenicity hypotheses, the participant outcomes for a trial arm with a safety decision rule (arm 1 in our trial design) were drawn from independent binomial distributions of the binary safety and immunogenicity endpoints respectively, and repeated in 10,000 simulation runs. We also performed ancillary simulation studies based on draws of participant outcomes from multinomial distributions, with specified joint probabilities of the two endpoints (corresponding to positive correlations from 0.1 to 0.5 between immunogenicity response and occurrence of vaccine-related toxicity events) [42], while keeping the marginal proportions of the endpoints constant. Recruitment dynamics were not taken into account for the simulation studies. Simulations were performed with R software, version 2.13.0 (The R Foundation, Vienna, Austria).

Furthermore, we projected the expected dynamics of recruitment into the trial and the gain in timelines of the clinical development plan by the proposed design based on our previous experience setting up and conducting HIV vaccine trials in France [32,43,44].

## Results and discussion

We adapted a randomised phase II design by including two different types of early safety decision rules. We first describe the characteristics of the proposed design with each safety decision rule and its practical implications. We then discuss potential alternative designs as well as potential limitations of our design and further perspectives.

### Characteristics of the proposed optimised phase I/II design

We propose a randomised four-arm phase I/II design with two independent binary endpoints for safety and immunogenicity, including a safety decision rule for vaccine 1 during the prime vaccination phase in arm 1 (Figure 1). The design is optimised in terms of a seamless transition from phase I to phase II and allows for a parallel, unbiased evaluation of several vaccine strategies in phase II.

The proportion of immunogenicity responders in each arm is assessed by a standardised interferon-gamma (IFN-γ) enzyme-linked immunospot (ELISPOT) assay at week 30, that is, 2 weeks after the last vaccine immunisation. There is a broad scientific consensus that a successful vaccine to prevent HIV transmission must be able to elicit both HIV-specific T-cells and antibodies responses [45,46]. Although previous research has shown that a positive IFN-γ ELISPOT response does not necessarily predict a protective vaccine effect against HIV acquisition [47], it is assumed that the IFN-γ ELISPOT response is at least a marker that the vaccine has some effect on the T-cells of the immune system. Under this assumption, the IFN-γ ELISPOT should allow to screen for vaccine immunogenicity and therefore seems adequate as a criterion to identify non-immunogenic vaccine strategies that do not elicit any cellular response [19]. Additional immunogenicity markers will be assessed as secondary endpoints.

Each arm of the phase II design has a single-stage Fleming design for the binary immunogenicity endpoint [48], comparing the proportion *P* of participants with a positive IFN-γ ELISPOT response to a theoretical unacceptable proportion of 50%. The randomised design with parallel arms is thus not intended for formal between-arm comparisons but to limit heterogeneity in volunteer characteristics between arms. In contrast with the randomised phase II selection designs used in oncology [14], this design does not aim to select one single strategy (that is, the ‘best’ strategy) at the end of the phase II design but retains all strategies with responses above a minimal level. The decision rule is formulated per arm and does not depend on the number of parallel arms in the trial, which distinguishes its operating characteristics from those of randomised phase II selection designs [49]. The sample size of n = 23 per arm was determined from published sample size tables based on exact binomial distributions, with 90% power, one-sided type I error rate of 5%, p1 of 80% (target proportion of IFN-γ ELISPOT responders), and p0 of 50% (unacceptably low proportion of IFN-γ ELISPOT responders) [50].

The integrated phase I evaluation of vaccine 1 is based on an independent binary safety endpoint at week 2 in arm 1, evaluating the proportion of participants without any grade 3 or 4 clinical or biological adverse events related to vaccine 1 immunisation, reported from week 0 to week 2. Event relatedness will be reviewed and grades validated by the Endpoint Review Committee.

We evaluated two types of safety decision rules, with the aim to avoid exposure of additional participants to an unsafe vaccine during the vaccine boost phase.

First, we considered a frequentist fixed-sample approach, which was also derived from a single-stage Fleming design, with 90% power, one-sided type I error rate of 5%, p1 of 95% (target proportion of participants without any vaccine-related adverse event), and p0 of 70% (unacceptably low proportion of participants without any vaccine-related adverse event). Based on the proportion at which the lower bound of the one-sided exact 95% confidence interval includes p0, this translates into the following safety decision rule for vaccine 1 [50]: if more than two out of the first 19 participants in arm 1 experience a vaccine-related adverse event by week 2, then all vaccine 1 injections will be stopped.

Second, we studied a Bayesian sequential safety monitoring approach, allowing (1) for formally incorporating the previously available knowledge about the safety of MVA vectors in the prior distribution, and (2) for continuously updating the posterior distribution after each participant or observed event during the trial [51]. We used an enthusiastic prior distribution (Beta (6, 0.3)) to reflect the sound prior knowledge about the safety of this vector type. The Bayesian safety decision rule was then defined based on the posterior probability that the proportion of participants without any vaccine-related adverse event is below the target proportion of 0.95. At any sequential analysis, the injections of vaccine 1 would be stopped if this posterior probability was greater than 95% (Prob (Psafe < 0.95|data) > 0.95).

Given the planned rapid accrual dynamics of the trial, the proposed safety decision rules do not aim at halting enrolment, which may be completed before the decision rule applies. Thus, the decision rule is not necessarily expected to impact the number of volunteers enrolled. Rather, it aims at taking a go/no-go safety decision before the start of the vaccination boost phase at week 20 to avoid exposing additional participants to vaccine 1 in the other trial arms. If administrations of vaccine 1 are stopped, arms 1 and 2 are halted but arms 3 and 4 continue, with participants in arm 4 receiving boost injections of vaccine 2 instead of vaccine 1.

Simulation results for each of the two safety decision rules are shown in Table 1.

**Table 1:** Comparison of operating characteristics of the two types of safety decision rules

**Type of safety decision rule**		**Simulation scenario**		
	**Vaccine safe (P**_**safe**_ **= 0.95)**	**Vaccine unsafe (P**_**safe**_ **= 0.7)**		
	**Probability of stopping**	**Probability of stopping**	**Participant number at stop (median; IQR)**	
Fixed-sample frequentist rule at n = 19	0.07	0.95	19	(19-19)^a^
Continuous Bayesian monitoring	0.05	0.96	8	(4-12)

Fixed-sample frequentist rule: Interim analysis after 19 participants. Vaccine considered unsafe at interim if lower bound of one-sided exact 95% confidence interval of observed proportion ≤0.7 for safety endpoint.

^a^In a strict application of the fixed sample-design the decision rule only applies when the outcome of 19 participants has been observed. If the application of the rule was handled more flexibly (that is stopping as soon as a third participant experiences an vaccine-related grade 3 or 4 adverse event, regardless of the denominator), stopping would occur after nine participants in median (IQR 6-12).

Continuous Bayesian monitoring of the safety endpoint: Sequential analysis after each participant or event. Vaccine considered unsafe at the first analysis where the posterior probability that the vaccine is below the target level (Psafe <0.95) exceeds 95%. Enthusiastic prior: beta (6,0.3).

Table 2 summarises the combined operating characteristics of a trial arm in the final phase I/II design, including both the frequentist analysis of the immunogenicity endpoint at week 30 as well as the continuous Bayesian monitoring of the safety endpoint at week 2. The simulated operating characteristics were satisfactory under all combinations of the hypotheses for safety and immunogenicity respectively, with a maximum overall error rate of 12.1%.

**Table 2:** Combined operating characteristics of the design including a frequentist efficacy evaluation and the continuous Bayesian safety decision rule

**Simulation scenario**		**Proportion of outcomes in 10,000 simulations of one trial arm (%)**			
**Safety hypothesis**	**Immunogenicity hypothesis**	**Vaccinations stopped (unsafe)**	**Vaccinations not stopped (safe), inefficacious at final**	**Vaccinations not stopped (safe), efficacious at final**	**Overall probability of erroneous conclusion**
Unsafe (0.70)	Inefficacious (0.50)	95.5	4.2	0.3	**4.5**
Unsafe (0.70)	Efficacious (0.80)	95.6	0.3	4.1	**4.7**
Safe (0.95)	Inefficacious (0.50)	4.8	91.4	3.8	**8.6**
Safe (0.95)	Efficacious (0.80)	5.3	6.8	87.9	**12.1**

Simulation methods: Simulation of independent binomial distributions for safety and efficacy, respectively. Simulation of participant outcomes for one trial arm with continuous Bayesian monitoring of the safety endpoint, repeated in 10,000 trial simulations per simulation scenario.

Bayesian continuous monitoring of the safety endpoint. Decision rule for safety outcome: Vaccine considered unsafe at the first analysis where the posterior probability that the vaccine is below the target level (Psafe <0.95) exceeds 95%. Enthusiastic prior: beta (6,0.3).

Fixed-sample frequentist analysis of immunogenicity endpoint with 23 participants: conclusion that vaccine strategy is efficacious at final analysis if lower bound of one-sided exact 95% confidence interval of observed proportion >0.5. Proportion of trials with erroneous outcomes shown in bold. The overall risk of erroneous conclusion corresponds to the sum of erroneous trial outcomes.

Equivalent simulations of the combined operating characteristics of the design including the frequentist fixed-sample safety decision rule showed relatively similar results, albeit with a slightly higher maximum overall error rate (overall probability of erroneous conclusion 14.2% for the scenario with simulated safety and efficacy proportions of 0.95 and 0.80, respectively).

Additional simulations for scenarios with an intermediate safety proportion of 80% and the fixed-sample frequentist decision rule or the continuous Bayesian decision rule showed probabilities of carrying the vaccine forward to the next step of the development plan in between those observed in the simulations with a safety level of 70% and 95%, respectively.

Introducing slight to moderate correlations between immunogenicity response at week 30 and vaccine-related adverse events at week 2 in the simulations did not change the operating characteristics. This is due to the fact that the endpoints are based on the marginal proportions, which were kept constant by design of the simulations.

### Practical implications of the proposed design

**Figure 2:**
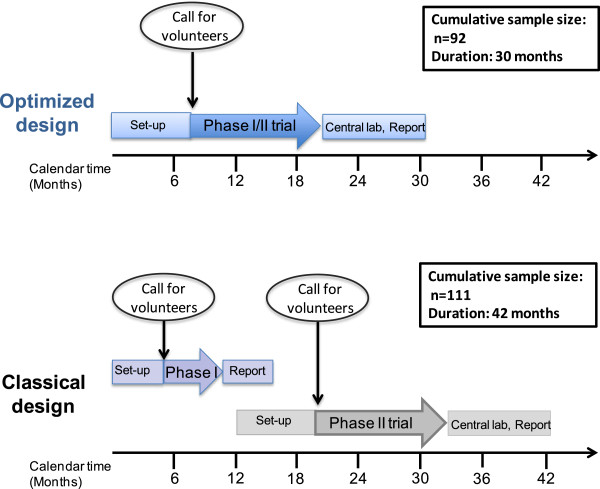
Gain in timelines with optimised phase I/II design compared to separate phase I and II trials.

As illustrated in Figure 2, compared with separate phase I and II trials, the proposed design with either safety decision rule allows for an estimated gain of approximately 12 months in the timelines of the clinical development plan.

In terms of trial logistics and data management, to be able to apply either of the decision rules in a timely manner, the proposed design requires real-time monitoring and validation of vaccine-related grade 3 and 4 adverse events until week 2.

The frequentist fixed-sample safety decision rule is stringent but requires high early accrual dynamics in order to have 19 evaluable participants at week 2 in arm 1 before any other participant reaches week 20 (first boost injection of vaccine 1). By using a continuous monitoring approach after each participant, the Bayesian rule allows for more flexibility and can be applied even if accrual dynamics deviate from the initial projections. This may for instance be the case if a certain time lag between the enrolment of the first couple of volunteers is desired in order to not expose several persons at once to a vaccine being first administered in human. Moreover, the Bayesian method allows for a direct interpretation of posterior probabilities.

Given the early safety decision rule, the standard frequentist estimate of the proportion of participants with severe vaccine-related adverse events throughout the whole follow-up in the trial (week 30), planned as a secondary safety endpoint, may be biased in the final analyses. If the final analysis stage is done from a frequentist perspective, specific methods for an unbiased estimation will thus need to be considered in order to take into account the fact that the final estimation is conditional on not stopping before [52].

### Alternative existing designs

Most alternative phase I or II designs available in the literature were not further considered due to the specific requirements for our trial design (see list of design requirements above and Table 3).

**Table 3:** Frequentist early-stage designs described in the literature and requirements for the vaccine trial design

		**Relevant features of the vaccine trial**						
		**Trial design and endpoints**					**Logistics**	
		**Absence of a validated surrogate endpoint for efficacy**^**a**^	**Efficacy outcome not assessed in real-time**^**b**^	**No control arm with ‘standard’ strategy or placebo**	**Possibility to move more than one strategy to the next development stage**	**Safety and efficacy endpoints at different time points**^**c**^	**High early accrual dynamics**^**d**^	**Randomised multi-arm multi-center trial**
**Potential alternative frequentist designs**								
**Type**	**Example**							
**A) Designs for both efficacy and toxicity evaluation**								
Non-comparative bivariate two-stage designs	Bryant and Day design [53]		No			No	No	
Seamless phase I/II design	Design proposed by Messer et al. (including a 3 + 3 design for the integrated phase I evaluation) [54]						No	No
**B) Toxicity stopping rules integrated in efficacy designs**								
Non-comparative stopping rule based on continuous toxicity monitoring per serious adverse event	Continuous monitoring proposed by Kramar et al. [55]						No	
Non-comparative stopping rule based on continuous toxicity monitoring per participant	Continuous monitoring proposed by Ivanova et al. [56]						No	
Non-comparative stopping rule based on group-sequential approach	Probabilistic approach proposed by Yu et al. [57]						No	
**C) Designs for efficacy evaluation, considered in combination with toxicity stopping rules in B)**								
Non-comparative two-stage or multi-stage designs	Gehan’s, Simon’s or Fleming’s design [48,58,59]		No				No	
Non-comparative treatment selection design	Ranking design by Simon [35]	No			No			
Comparative multi-arm designs	Comparative phase II designs; screening designs [60]	No		No				
	Group sequential designs; adaptive designs with comparative decision rule [61]	No	No				No	

Non-exhaustive list of trial features and alternative frequentist designs. Only main features of vaccine trial leading to incompatibility with alternative designs are indicated.

No: characteristic of the vaccine trial not compatible with alternative design.

The term ‘non-comparative’ is used to indicate that no inter-arm comparison is required.

^a^No single validated endpoint in HIV vaccine immunogenicity trial, since correlates of vaccine protection are unknown. Currently, multiple different immunogenicity measurements are of interest without any definite hierarchical order in their relevance (multidimensional data) and no obvious definition of a composite endpoint. In the present vaccine trial, the primary immunogenicity endpoint is only used as a screening assay to discard out non-immunogenic strategies.

^b^Immunogenicity measurements done in batch on frozen samples at the end of the trial.

^c^Safety evaluation at week 2; Efficacy evaluation at week 30.

^d^High early accrual dynamics expected after a single call for volunteers in the media.

We gave thorough consideration to alternative designs to evaluate the safety of vaccine 1 within a phase II trial but ruled them out for the following reasons:

Continuous toxicity monitoring approaches from the frequentist perspective, such as those suggested by Goldman et al., Kramar et al., Ivanova et al. or Ray et al. [42,55,56,62] require that the participants (or events) be observed sequentially (that is, one by one), which is not granted with high accrual dynamics. An alternative approach to Bayesian continuous safety monitoring could have been designed based on the predictive probability instead of the posterior distribution. In the context of efficacy monitoring with slightly higher maximum sample sizes than in our design it has been shown to have similar operating characteristics than the method based on the posterior distribution [63]. However, applying decision rules based on future probabilities to a safety endpoint is conceptually less intuitive, and we believe that a rule based on the current posterior distribution better resembles the decision making process for safety. For the same rationale, we did not pursue the approach published by Yu et al. who suggested toxicity monitoring based on a probabilistic approach estimating the future rate of toxicity [57].

Most existing bivariate two-stage designs for combined safety and efficacy evaluation, be they frequentist or Bayesian [53,64-67], were developed in the context of cytotoxic cancer drug development and generally require both the efficacy and the safety outcome to be observed and analysed with the same timing. This is not the case in our vaccine trial where the immunogenicity outcome is measured in batch at the end of the trial. Although a group-sequential bivariate design with increased flexibility, accommodating different schedules for efficacy and safety analyses was also suggested, this design still requires at least one analysis time including both safety and efficacy endpoints [68].

Lastly, we considered the seamless phase I/II frequentist design proposed by Messer et al. [54], the rationale of which is very close to ours. However, their design is appropriate for a single-arm trial, using a 3 + 3 enrolment scheme in phase I, but is too complex to implement in the context of a multi-center multi-arm trial and is not compatible with the accrual dynamics expected for the healthy volunteers in our trial. Other seamless phase I/II designs comprising a dose-finding phase have the same limitations for application in our context [69].

### Limitations of the proposed design

The design we propose fulfils all our specified requirements but has some limitations.

First, the set-up of a combined phase I/II design is resource-efficient under the assumption that vaccine 1 is safe, but is less efficient if vaccine 1 injections were stopped for insufficient safety. However, even if this is the case, two arms will still continue the trial (arms 3 and 4, then both receiving the same vaccine strategy with vaccine 2 as boost injections and merged together for the final analysis). This will double the number of participants receiving the strategy with vaccine 3 prime and vaccine 2 boost and result in increased precision for the non-comparative statistical analysis of this strategy. Thus, even if vaccine 1 is stopped, at least some resource-efficiency of the proposed four-arm trial will be maintained. However, the proposed design would be less efficient in a context, in which the safety record of the vaccine candidate is more uncertain.

Second, the proposed design does not include a further transition into the next development phases, and a separate phase II follow-up trial will be necessary to evaluate the immunogenicity spectrum of the vaccine strategies with more precision.

Third, albeit randomised, our trial is not designed for inter-arm comparisons. Investigators should be careful about making indirect comparisons of the point estimates in the different arms without considering precision [70].

Lastly, no multiplicity adjustment is planned in our design to account for the multiple trial arms. However, the control of false-positive selection for immunogenicity (type I error) is not a major concern in this early-stage trial, since independent follow-up trials are scheduled in the clinical development plan.

### Further perspectives in the clinical development plan

Set-up of a HIV vaccine trial using the design with the Bayesian safety decision rule and including a 24-hour lag between the enrolment of the first 20 volunteers is currently ongoing in a protocol of the French Vaccine Research Institute (VRI).

At the end of this trial it is planned to carry all immunogenic and safe vaccine strategies forward to further evaluations in another phase II immunogenicity trial, since more data should be collected before deciding to set up a large scale phase IIB/III trial with an HIV-acquisition endpoint. However, unless the knowledge about immunological correlates of protection and surrogate makers evolves substantially, it will remain difficult to predict protective vaccine efficacy on the basis of phase II immunogenicity results and the decision to move the clinical development to a phase IIB/III trial will be delicate. A group-sequential phase IIB/III design could thus be envisaged, with interim looks at the HIV-acquisition endpoint to allow for early stopping of strategies for lack of efficacy or harm [17].

## Conclusions

We used a pragmatic approach to design an optimised randomised phase I/II trial for the evaluation of the safety and immunogenicity of several HIV vaccine strategies. The design can include either a frequentist fixed-sample or Bayesian continuous early safety decision rule and allows for data integration of phases I and II for the final analysis. Therefore, acceleration of early clinical development of HIV vaccine strategies is, to some extent, possible, but requires thoughtful planning at the design stage. Validation of surrogate markers for HIV vaccine efficacy will be crucial for the implementation of more complex adaptive phase II designs.

During the HIV vaccine development process, candidate vaccines are often improved in terms of the antigen inserts used. The situation where a well-known vector with a modified insert is administered first in humans without dose escalation is thus not unusual in HIV vaccine development portfolios. Similar thoughts may also apply to vaccine research for other diseases requiring complex vaccine strategies, for example, malaria vaccines. Although some aspects discussed in this article are specific to HIV vaccine research, the suggested early-phase design could therefore also be useful for the clinical development of other complex vaccine portfolios with similar methodological requirements.

## Abbreviations

DNA: Deoxyribonucleic acid; ELISPOT: Enzyme-linked immunospot; HIV: Human immunodeficiency virus; Inserm-ANRS: Institut National de la Santé et de la Recherche Médicale-Agence Nationale de Recherches sur le Sida et les Hépatites Virales (French National Agency for Research on AIDS and Viral Hepatitis); MVA: Modified vaccinia Ankara; IFN-γ: Interferon-gamma; IQR: Interquartile range; VRI: Vaccine Research Institute; W: Week.

## Competing interests

The institutions of LR, JDL, VA, YL, GC and RT receive grants from the Vaccine Research Institute (VRI) and from the French National Agency for Research on AIDS and Viral Hepatitis (Inserm-ANRS). VR and AB are employees of Inserm-ANRS. The authors have no other competing interests.

## Authors’ contributions

LR designed the trial, developed and implemented the methods, performed the literature review, performed and interpreted the simulation studies, participated in the implementation of the trial and led the drafting of the manuscript. AD participated in the design of the trial, interpreted the simulation studies, and contributed to the literature review and to the writing of the manuscript. JDL designed, implemented and coordinates the trial, and contributed to the writing of the manuscript. VA, VR and AB participated in the implementation and coordination of the trial, and contributed to the writing of the manuscript. YL participated in the design of the trial and contributed to the writing of the manuscript. GC participated in the design, implementation and coordination of the trial, interpreted the simulation studies, and contributed to the writing of the manuscript. RT designed the trial, developed the methods, interpreted the simulation studies, and contributed to the literature review and to the writing of the manuscript. All authors read and approved the final manuscript.

## ANRS VRI 01 protocol team

VRI Clinical Core: Jean-Daniel Lelièvre and collaborators (Groupe Henri-Mondor Albert-Chenevier, Créteil); Frédéric Lucht and collaborators (CHU, St Etienne); Odile Launay and collaborators (Hôpital Cochin, Paris); Isabelle Poizot-Martin and collaborators (CIC-UPCET, Marseille); Dominique Ducard, Dominique Maingueneau, Caroline Ollivier-Yaniv (UPEC, Créteil); Vincent Arnold, Florence Allais, Geneviève Chêne, Catherine Fagard, Lucile Hardel, Adélaïde Perrier, Helen Savarieau (Centre INSERM U897, Bordeaux).

VRI Biostatistics Core: Laura Richert, Rodolphe Thiébaut (Centre INSERM U897, Bordeaux).

VRI Immunology Core: Christine Lacabaratz, Yves Lévy, and collaborators (INSERM U955, Créteil).

Inserm-ANRS: Amel Bouakane, Alpha Diallo, Véronique Rieux (Inserm-ANRS, Paris).
